# The Evaluation of Physical Stillness with Wearable Chest and Arm Accelerometer during Chan Ding Practice

**DOI:** 10.3390/s16071126

**Published:** 2016-07-20

**Authors:** Kang-Ming Chang, Yu-Teng Chun, Sih-Huei Chen, Luo Lu, Hsiao-Ting Jannis Su, Hung-Meng Liang, Jayasree Santhosh, Congo Tak-Shing Ching, Shing-Hong Liu

**Affiliations:** 1 Department of Photonics and Communication Engineering, Asia University, 500, Lioufeng Rd., Wufeng, Taichung 41354, Taiwan; changkm@asia.edu.tw; 2 Department of Medical Research, China Medical University Hospital, China Medical University, No. 91, Hsueh-Shih Road, Taichung 40402, Taiwan; 3 Biosignal Processing Lab, Asia University, 500, Lioufeng Rd., Wufeng, Taichung 41354, Taiwan; ken7360@gmail.com.tw (Y.-T.C.); abbykayq@yahoo.com.tw (S.-H.C.); 4 Department of Business Administration, National Taiwan University, No. 1, Sec. 4, Roosevelt Rd., Taipei 10617, Taiwan; luolu@ntu.edu.tw; 5 Department of Educational management and law, National Taipei University of Education, No.134, Sec. 2, Heping E. Rd., Da-an District, Taipei 10671, Taiwan; jannis56789@yahoo.com.tw; 6 Department of Law, National Chung Cheng University, No.168, Sec. 1, University Rd., Min-Hsiung Township, Chia-yi County 62102, Taiwan; lawlhm@ccu.edu.tw; 7 Department of Biomedical Engineering Faculty of Engineering, University Malaya, Jalan Elmu, Off Jalan University, Kuala Lumpur 59100, Malaysia; jsanthosh@um.edu.my; 8 Department of Electrical Engineering, National Chi Nan University, Daxue Rd., Puli Township, Nantou County 545, Taiwan; 9 Department of Computer Science and Information Engineering, Chaoyang University of Technology, 168, Jifeng E. Rd., Wufeng District, Taichung 41349, Taiwan

**Keywords:** Chan Ding, wearable accelerometer, physical stillness index (PSI), meditation, Chinese Happiness Inventory

## Abstract

Chan Ding training is beneficial to health and emotional wellbeing. More and more people have taken up this practice over the past few years. A major training method of Chan Ding is to focus on the ten Mailuns, i.e., energy points, and to maintain physical stillness. In this article, wireless wearable accelerometers were used to detect physical stillness, and the created physical stillness index (PSI) was also shown. Ninety college students participated in this study. Primarily, accelerometers used on the arms and chest were examined. The results showed that the PSI values on the arms were higher than that of the chest, when participants moved their bodies in three different ways, left-right, anterior-posterior, and hand, movements with natural breathing. Then, they were divided into three groups to practice Chan Ding for approximately thirty minutes. Participants without any Chan Ding experience were in Group I. Participants with one year of Chan Ding experience were in Group II, and participants with over three year of experience were in Group III. The Chinese Happiness Inventory (CHI) was also conducted. Results showed that the PSI of the three groups measured during 20–30 min were 0.123 ± 0.155, 0.012 ± 0.013, and 0.001 ± 0.0003, respectively (*p* < 0.001 ***). The averaged CHI scores of the three groups were 10.13, 17.17, and 25.53, respectively (*p* < 0.001 ***). Correlation coefficients between PSI and CHI of the three groups were −0.440, −0.369, and −0.537, respectively (*p* < 0.01 **). PSI value and the wearable accelerometer that are presently available on the market could be used to evaluate the quality of the physical stillness of the participants during Chan Ding practice.

## 1. Introduction

Nowadays, wearable devices can detect an abundant amount of physiological information, such as heart rate, temperature, etc. Activity recognition can be measured using wearable sensor devices [1]. Accelerometers are broadly used in many applications, such as Nintendo Wii [2,3], Apple watches [4], other sport watches [5], and telemedicine healthcare systems [6]. The function of an accelerometer is to measure acceleration forces. [7]. Marschollek measured physical activity patterns, and identified them using wearable sensor devices [8]. Wearable devices are also widely used for health care services [9]. For medical treatment, phones and wearable systems are well integrated and broadly applied in a variety of motion detections, and sensor networks are used extensively in the detection of daily activities [10,11], such as fall detection [12,13], gait analyses [14], quality of sleep [15], energy expenditure [16], physical therapy exercises [17], classifying human activities [18], and even everyday acts in model tracking, such as the actions of people who suffer from Parkinson’s disease [19]. All these activities have one thing in common: They detect dynamic activities. However, only some research has studied the topic of physical stillness. The detection of physical stillness is the opposite concept of dynamic activities. Some clinical studies have researched physical stillness in autism spectrum disorder [20] and attention deficit hyperactivity disorder [21]. Researchers wanted to know that children how to maintain stillness of the body. In the practice of Chan Ding, the body must be kept still; therefore, the practice of Chan Ding can be evaluated by detecting the movements of the body. If people can keep their bodies still while practicing Chan Ding, it shows that they are well trained in this technique.

Many studies have investigated the benefits of meditation. One of the most distinguished research groups is from Davidson’s lab. Davidson et al. investigated the function of brain dynamics during meditation [22]. Their studies revealed that meditation increases brain activity and function [23]. Meditation can allow people with cardiovascular sequelae to quickly change their emotional state from negative to positive [24], and may even alter brain waves [25]. Similar research on Chan Ding addressed the benefits of relieving an individual’s stress [26]. In order to meditate effectively, the Chan Ding approach is considered as one of the most effective by normal people. Chan Ding originated from Sakyamuni Buddha, 2500 years ago, and has now attracted more than 100,000 people who practice it in Taiwan. If people practice Chan Ding, they are supposed to learn to attain physical stillness and to pay attention to their energy points (called Mailuns or chakras). In this way, it is aimed to help the practitioners to explore the inner peace and to gain health, wisdom and energy [27]. Chan Ding practice is even supposed to be able to regulate autonomic activities like blood pressure etc. to allow the body to feel relaxed and have less anxiety [28].

According to the research of Pavot et al., happiness is “a wide range of phenomena with positive emotional presentation instead of negative emotion” [29]. People with a high sense of well-being will not be involved in unhealthy habits behaviors, such as smoking, poor diet, and alcohol/drug abuse [30]. Ramesh et al. investigated the relationship between meditation and happiness by using the Oxford Happiness Questionnaire. They pointed out that there were significant differences between meditation groups’ and non-meditation groups’ sense of happiness [31]. The definitions of happiness are very varied, depending on different cultures. Luo has applied nine sources of happiness to the Chinese culture, which are as follows: (a) need for respect; (b) harmony of interpersonal relationships; (c) satisfaction of material needs; (d) achievements at work; (e) being at ease with life; (f) being appreciative of other’s devotions; (g) sense of self-control and self-actualization; (h) pleasure and positive influence; and (i) health. He developed the “Chinese Happiness Inventory” (CHI) as an effective measurement for Chinese’ happiness [32].

One of the basic hypotheses of Chan Ding is that practicing people experience a feeling of happiness by fully focusing on the Mailuns without any movement of the body. Therefore, it is interesting to investigate the relationship between the physical stillness index (PSI) values and happiness scores occurring during Chan Ding practice. In a previous study [33], a wireless accelerometer was used to detect physical stillness during meditation. An experienced group and a beginner group had significant differences. Other research regarding wearable sensors and long-term activity measurements were shown in references [34,35]. In this article, we noted that where the wearable accelerometer was placed had a higher sensitivity in detecting body movements. Then, the physical stillness of the participant during Chan Ding practice was investigated using a wireless accelerometer on the arm. The purpose of this study was to establish a PSI that could be used to evaluate the quality of Chan Ding practice. The relationship between the CHI scores and PSI values for the different Chan Ding experience-level groups were also investigated.

## 2. Methodology

### 2.1. Participants

Ninety participants were enrolled in this experiment. Their ages ranged from 18 to 30. Participants were divided into three groups, and each group had 30 participants. Group I was the inexperienced group: The male/female ratio was 9/21, with an average age of 23.4 with standard deviation (std) 3.8 years. Group II was the beginner group, who had practiced Chan Ding for less than one year: The male/female ratio was 10/20, with an average age of 20.6 with std 1.2 years. Group III was the advanced group, having practiced Chan Ding for more than three years: The male/female ratio was 10/20, with an average age of 22.8 with std 6.8 years. This experimental process was approved by the Asia University Medical Research Ethics Committee (Number 10211001).

### 2.2. Surveying Instruments

Two types of accelerometers were used in the following measurement. The first wireless accelerometer is the TD1A system (K&Y Lab, Taipei, Taiwan). It has a tri-axis accelerometer and its specifications are 0.73 G/cm, measures 50 × 30 × 10 mm, it weighs 11 g, and the sampling frequency is 500 Hz. The second wireless accelerometer is the Monnit wireless accelerometer (Monnit Corporation, Murray, UT 84123, USA). Its size is 26.4 × 45 × 19 mm and its weight is 12 g. The data is displayed in degrees with 0.1° of resolution. The short version of the “Chinese Happiness Inventory”, designed by Lu et al., was used [36]. There are 20 items in the survey. The survey’s Cronbach alpha value was 0.92, which shows high reliability and validity.

### 2.3. The Experiment of Wearable Accelerometers

**Figure 1:**
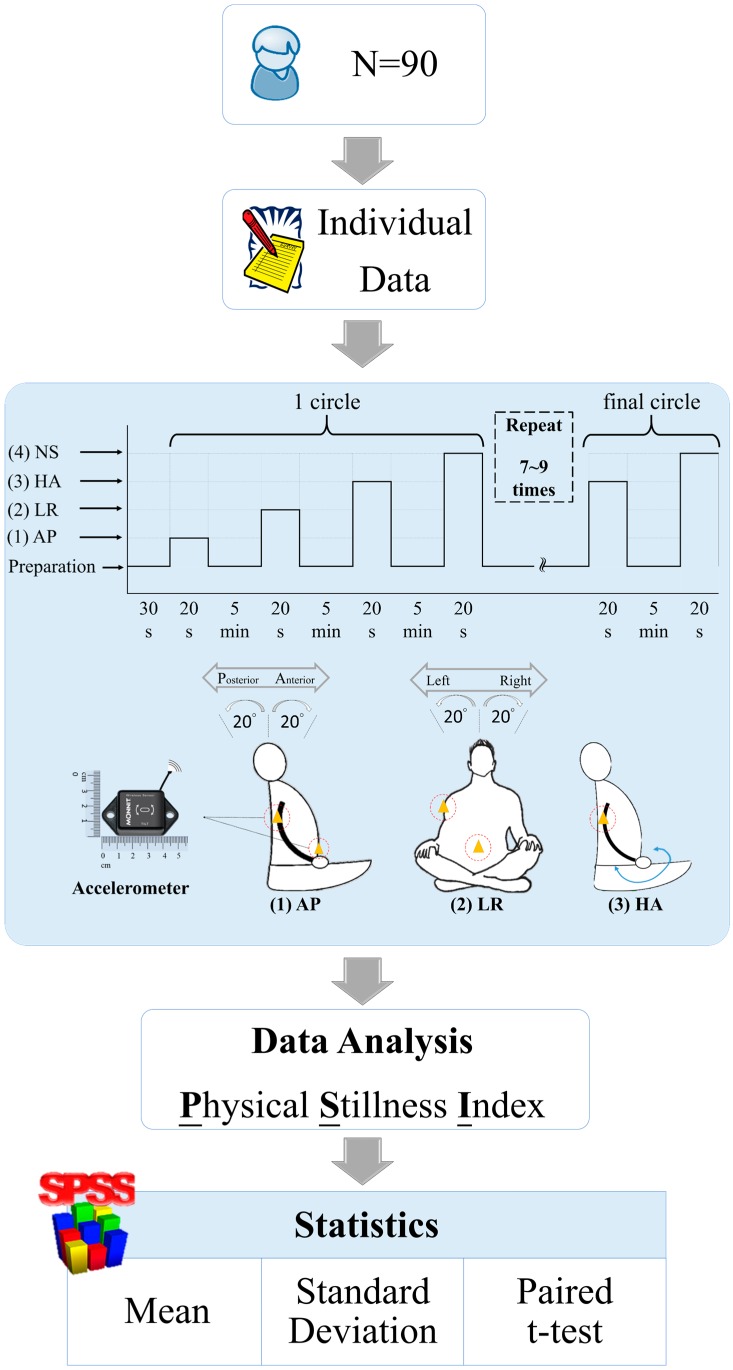
Flowchart of the first experiment.

**Figure 2:**
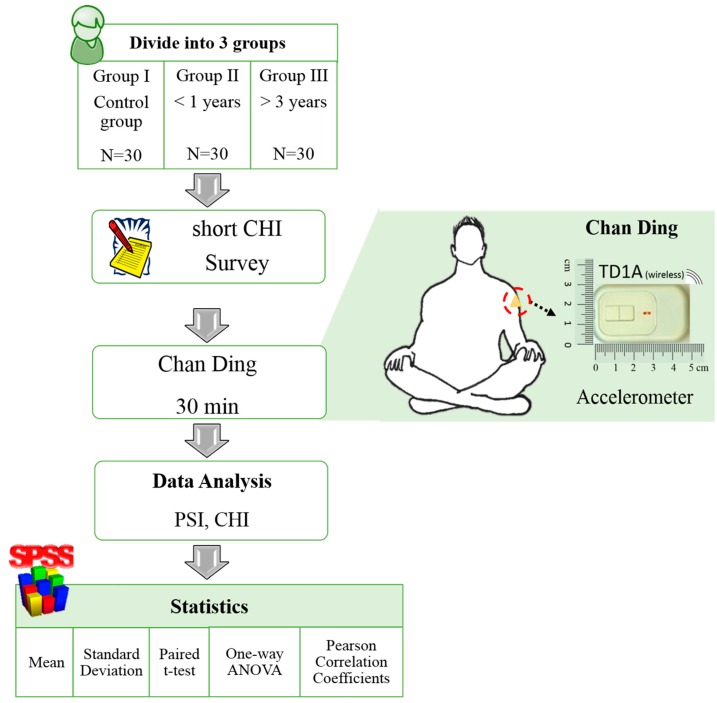
Flowchart of the second experiment.

There were two experiments in this study. The first experiment was to identify the signals of the various body movements during Chan Ding practice. Three accelerometers were worn on both upper arms and on the chest (xiphoid) to measure body movement signals, as shown in Figure 1. We defined four kinds of movements: (1) anterior-posterior movement was denoted as AP; (2) left-to-right movement was denoted as LR; (3) hand movement was denoted as HA; (4) no swaying with natural breathing was denoted as NS. The periods of AP and LR were four seconds long, and the period of HA was two seconds. The movement angles of AP and LR were 20°, 40°, and 60°. In one cycle, the sequence of body movements was AP, LR, HA and NS. Each activity lasted about twenty seconds. The resting time between activities was 5 min. In this experiment, participants were asked to repeat the four movement types seven or eight times. The second experiment was to measure PSI distribution during the practice of Chan Ding. One accelerometer was worn on the upper arm. Participants practiced Chan Ding for more than 30 min, as shown in Figure 2.

### 2.4. Physical Stillness Index (PSI)

PSI is defined according to the previous research [33]. First, the base lines were subtracted from the three measured axes signals of the accelerometer:

(1) $$\tilde{X_{i}\left[n\right]}=X_{i}\left[n\right]-\mathrm{mean}(\{X_{i}\left[n\right]\})\;i\;=1,\;2,\;3$$

where X is the signal of the accelerometer for one axis, *i* represents the X, Y, or Z axis, {X*_i_*[n]} represents the set for one measured set of data.

(2) $$S_{1}\left[n\right]=\overset{3}{\underset{i=1}{\sum }}X_{i}^{2}\left[n\right]\;i=1,\;2,\;3$$

(3) $$S_{2}\left[n\right]=\sqrt{\frac{1}{2N+1}\overset{N}{\underset{j=-N}{\sum }}S_{1}\left[n+j\right]},$$

where N = 10.

PSI = mean({S_2_[n]}) (4)

where {S_2_[n]} represents the set for S_2_.

### 2.5. Data Analysis and Statistics

We employed the SPSS 12.0 software package to conduct data analyses [33]. Descriptive statistics were used to show the parameter distribution as the mean ± standard deviation (std). A paired *t*-test was used to examine the differences of the sensor locations. One-way analysis of variance (ANOVA) was used to examine the differences in PSI among the three groups [37]. Pearson correlation coefficients between the PSI values and the CHI scores were also evaluated.

## 3. Result

### 3.1. The PSI Analysis for Body Movements

Table 1 shows the PSI results for experiment 1. We found that the PSI of the upper arm was significantly higher than that of the chest for any angle of the LR vibrations. For the AP vibrations, only the PSI of the upper arm, under 20° vibration, had no significant difference to that on the chest. Moreover, the PSIs of the upper arms all had significant differences with that of the chest for HA and NS movements. The average PSI values of the upper arm were, on average, greater than the PSI values for the chest. The average PSI ratios of arm to chest are around 1.1 to 4.6. These are AP (40°), AP (60°), LR (20°), LR (40°), LR (60°), and HA. These results show that the accelerometer worn on the upper arm had a higher sensitivity to detect body movement.

### 3.2. Long-Term Chan Ding PSI Analysis

Table 2 shows the average PSI values of the three groups for experiment 2. There were significant differences among the three groups. For experienced Chan Ding participants, their PSI values were lower than those of the other two groups, and below 0.001 g for thirty minutes. Participants in group I performed with an average PSI of around 0.013 g in the first 10 min, which is greater than Group III. The ranks of these three groups’ averaged PSIs were as follows: Group I ranked number one, Group II ranked number two, and Group III ranked number three. Further comparative analysis found that the PSI of Group I was also significantly greater than that of Group II and Group III for the different times used. Nonetheless, no significant differences were demonstrated between Group II and Group III.

### 3.3. Chinese Happiness Inventory Score and PSI

Table 3 shows the CHI scores of the three groups and the correlation coefficients between the PSI values and CHI scores. The average CHI scores were: Group I (10.13) < Group II (17.16) < Group III (25.53), with a significant difference of (*p* < 0.001). The difference of the PSIs among each of the two groups was also significant. There were also strong negative correlations between the CHI scores and the PSI values among the three groups. This suggests that the experienced Chan Ding practitioners obtained a higher degree of CHI scores, and they also had a lower PSI value during Chan Ding practice.

## 4. Discussion

In a previous experiment [33], the main study was regarding utilizing an accelerometer to investigate common changes of posture when practicing Chan Ding. For instance, different positions of the legs, or body movements, may move the center of gravity in a different direction. In this study, in order to find where the optimal placed position was for the accelerometer to detect the PSI, participants wore accelerometers on their arms and chest. In Table 1, the results show that the PSI values of the arms and chest had statistically significant differences with almost every movement. When breathing naturally, the PSI values of the arms and chest were equal. Therefore, the arm is an optimal place to wear an accelerometer to measure PSI. For long-term comparison among the three groups, the body movements of participants, during Chan Ding practice, were measured, as shown in Table 2. Significant differences were found between the inexperienced group, the beginners group, and the advanced group. The PSI value of the inexperienced group was a much greater than that of the beginner group. The PSI value of the beginner group was greater than that of the advanced group.

Participants in the Chan Ding practice have to remain stable, overcome the pain in their legs, pay attention to their breathing, and concentrate on the inner invisible ten-Mailuns simultaneously as well. After practicing Chan Ding for several months, many participants feel frustrated and give up on practicing. A proper measurable index to inform them of their range of improvement would be beneficial to their motivation. As shown in this article, the PSI value between experienced Chan Ding participants and inexperienced participants were obviously different after only 20–30 min. Data from Table 2 also indicate that the average PSI ratio between Group III and Group I during the three phases were 13, 31, and 123, respectively. Similar ratios derived from Table 3 in a previous study [33] were 1.67, 1.61, and 1.51, respectively. This study demonstrated that an accelerometer on the arm is more sensitive than one on the chest to detect PSI. Therefore, Chan Ding participants can see the measurable PSI to evaluate the quality of meditation rather than abstract mental feelings. This can motivate Chan Ding participants to practice.

The sense of happiness is very subjective; after Chan Ding training, participants felt healthier and happier. As shown in this experiment, the sense of happiness among these three groups scored as significantly different. The scores of the advanced group ranked at number one; the scores of the beginners ranked at number two; and the scores of the Chan Ding group without experience ranked at number three. As shown in the correlation analysis between the PSI values and the CHI scores, in Table 3, their relationship is significantly negative. That means that the lower the PSI values were, the higher the scores of happiness would be. Participants felt happier when their PSIs were lower. Because the CHI score lacks complete norms for the scale in this study, this research used the results of previous experiments to compare. In the unpublished thesis of Shu-huei Lin, 782 freshmen, studying at a Taiwanese University, participated in completing the 10-question CHI scale. Total scores were 30 points and the average score was 11.7 points [38]. This result was consistent with the average score from Group I (10.13). The average happiness scores of Group II and III were 17.17 and 25.53, respectively. The higher scores in Group II and Group III indicated that practicing Chan Ding has an obvious benefit for mental health. The discussion of the mental health benefits of meditation was presented in the previous study [38] and the SF36 questionnaire was used. According to an Australian health article, the mental health scores of long-term meditation participants were 85 points. The score of other Australians, without experience in Chan Ding, were 76 points [39]. In addition, after eight weeks of meditation practice, the mental health scores of 99 participants with chronic pain, ranged from 55.66 points to 67.4 points [40]. The Beck Anxiety Inventory scale is frequently utilized to measure differences after meditation practice; after eight weeks of meditation practice, the anxiety scores of patients suffering from anxiety disorders ranged from 21.41 to 8.29 [41]. These experimental results were consistent with those in the present study. This study was the first time that CHI has been applied to local subjects. The longer the participants received Chan Ding training, the better the stability of their bodies and sense of happiness were. To the authors’ knowledge, no experiments have been conducted in this area before. This research attempted to connect the data between many questionnaires of mental health and physical movement in order to investigate body posture stability by use of the PSI. Recently, accelerometer combined with wearable devices, e.g. cell phones was used to detect physical activities [42,43,44]. Those wearable devices have the potential to be used in the future as auxiliary instruments to facilitate Chan Ding beginners to improve the quality of their body posture stability.

## 5. Conclusions

This research verified that an accelerometer on the arm can measure the body’s stable index of the participant during Chan Ding practice effectively and keenly. It can prevent breathing intervention as well. This testing method performed better than a previously investigated method in which a sensor was placed on the chest and belly.

## Figures and Tables

**Table 1:** The PSI results of the first experiment. Data is represented as mean ± STD (unit is g, gravitation).

Body Movement	PSI of the Chest	PSI of the Upper Arm	*p*-Value	Average Ratio of Arm to Chest
AP (20°)	0.09 ± 0.06	0.10 ± 0.08	0.61	1.1
AP (40°)	0.17 ± 0.08	0.23 ± 0.10	<0.001 ***	1.4
AP (60°)	0.24 ± 0.07	0.38 ± 0.09	<0.001 ***	1.6
LR (20°)	0.06 ± 0.03	0.09 ± 0.05	<0.01 **	1.5
LR (40°)	0.14 ± 0.06	0.16 ± 0.07	<0.05 *	1.1
LR (60°)	0.20 ± 0.07	0.30 ± 0.11	<0.001 ***	1.5
HA	0.05 ± 0.03	0.23 ± 0.10	<0.001 ***	4.6
NS	0.01 ± 0.01	0.01 ± 0.01	<0.001 ***	1.0

* *p* < 0.05, ** *p* < 0.01, *** *p* < 0.001.

**Table 2:** The PSI values of the three groups with 3 different times. Data is represented as mean ± SD (unit is g, gravitation).

	Group I	Group II	Group III	*p*-Value
0~10 min (Phase I)	0.013 ± 0.018	0.004 ± 0.008	0.001 ± 0.001	0.001 **
10~20 min (Phase II)	0.031 ± 0.042	0.005 ± 0.008	0.001 ± 0.0005	<0.001 ***
20~30 min (Phase III)	0.123 ± 0.155	0.012 ± 0.013	0.001 ± 0.0003	<0.001 ***

** *p* < 0.01, *** *p* < 0.001 (Group I is the inexperienced group, Group II is the beginners group, Group III is the advanced group).

**Table 3:** Distribution of the Chinese Happiness inventory (CHI) scores in the three groups.

Item	Group I	Group II	Group III
CHI	10.13 (3.32) ^a^	17.17 (1.73) ^b^	25.53 (2.09) ^c^
Correlation coefficients between PSI/CHI score	−0.440 ^d^	−0.396 ^d^	−0.537 ^d^

^a^ *p*-value Group I vs. Group II < 0.001 ***; ^b^ *p*-value Group II vs. Group III < 0.001 ***; ^c^ *p*-value Group I vs. Group III < 0.001 ***; ^d^ *p*-value < 0.01 **.
